# Immune restoration disease and changes in CD4+ T-cell count in HIV- infected patients during highly active antiretroviral therapy at Zewditu memorial hospital, Addis Ababa, Ethiopia

**DOI:** 10.1186/1742-6405-7-46

**Published:** 2010-12-21

**Authors:** Kahsay Huruy, Afework Kassu, Andargachew Mulu, Yemataw Wondie

**Affiliations:** 1 Department of Medical Laboratory Technology, College of Medicine and Health Sciences, University of Gondar, Ethiopia; 2 Institute of Virology, Faculty of Medicine, University of Leipzig, Germany; 3 Department of Microbiology and Parasitology, College of Medicine and Health Sciences, University of Gondar, Ethiopia; 4 Division of Allergy and Clinical Immunology, Department of Medicine, University of Colorado, Denver, USA; 5 Faculty of Social Sciences and Humanities, University of Gondar, Gondar, Ethiopia; 6 Institute of Psychology II, Clinical and Health Psychology, University of Leipzig, Germany

## Abstract

**Background:**

Highly active antiretroviral therapy (HAART) improves the immune function and decreases morbidity, mortality and opportunistic infections (OIs) in HIV-infected patients. However, since the use of HAART, immune restoration disease (IRD) has been described in association with many OIs. Our objective was to determine the proportion of IRD, changes in CD4+ T-cell count and possible risk factors of IRD in HIV-infected patients.

**Methods:**

A retrospective study of all HIV- infected patients starting HAART between September 1, 2005 and August 31, 2006 at Zewditu memorial hospital HIV clinic, Addis Ababa, Ethiopia was conducted. All laboratory and clinical data were extracted from computerized clinic records and patient charts.

**Results:**

A total of 1166 HIV- infected patients with mean ± SD age of 36 ± 9.3 years were on HAART. IRD was identified in 170 (14.6%) patients. OIs diagnosed in the IRD patients were tuberculosis (66.5%, 113/170), toxoplasmosis (12.9%, 22/170), herpes zoster rash (12.9%, 22/170), *Pneumocystis jirovecii *pneumonia (4.1%, 7/170), and cryptococcosis (3.5%, 6/170). Of the 170 patients with IRD, 124 (72.9%) patients developed IRD within the first 3 months of HAART initiation. Low baseline CD4+ T-cell count (odds ratio [OR], 3.16, 95% confidence interval [CI], 2.19-4.58) and baseline extra pulmonary tuberculosis (OR, 7.7, 95% CI, 3.36-17.65) were associated with development of IRD. Twenty nine (17.1%) of the IRD patients needed to use systemic anti-inflammatory treatment where as 19(11.2%) patients required hospitalization associated to the IRD occurrence. There was a total of 8 (4.7%) deaths attributable to IRD.

**Conclusions:**

The proportion and risk factors of IRD and the pattern of OIs mirrored reports from other countries. Close monitoring of patients during the first three months of HAART initiation is important to minimize clinical deterioration related to IRD.

## Background

Highly active antiretroviral therapy (HAART) improves the immune function and decreases morbidity, mortality and opportunistic infections (OIs) in HIV-infected patients [1,2]. However, the introduction of HAART presents new clinical problems, including adverse drug effects, and the event of diseases that are as the result of the restoration of the immune response. When clinical deterioration occurs during immune recovery and is associated with the host inflammatory response to pathogens, the clinical presentation has been described as immune restoration disease (IRD), immune reconstitution inflammatory syndrome or immune reconstitution disease[3,4].

IRDs usually occur within a few weeks to months after the initiation of HAART and majority of patients with IRD present with unusual manifestations of OIs, most often while the number of CD4+ T lymphocytes is increasing and the viral load is decreasing [5,6]. Even if no consistent definition exists for IRD, its diagnosis requires the worsening of a recognized (paradoxical) or unrecognized (unmasking) pre-existing infection in the setting of improving immunologic function [7].

Previous studies of IRD in association with initiations of HAART to treat HIV infection differ widely and reports have indicated IRD ranges from 10-30% in patients who started HAART [8-10]. Majority of IRDs described in adults are commonly reported in association with *Mycobacterium tuberculosis *(MTB), a major cause of morbidity and mortality among patients living with HIV/AIDS worldwide [11,12]. IRD has also been associated with a range of OIs, including cytomegalovirus, hepatitis B and C viruses, *Pneumocystis jirovecii*, *Cryptococcus neoformans*, herpes viruses, progressive multifocal leucoencephalopathy, leishmaniasis, and cerebral toxoplasmosis [12].

In Ethiopia, antiretroviral therapy (ART) has been made available to HIV/AIDS patients since 2004. Although over 250,000 HIV/AIDS patients require ART in the country, only 24% of the eligible adults were on ART by the end of 2007 [13]. Studies on IRDs and Changes in CD4+ T-cell count among HIV- infected patients during HAART in Ethiopia are very scarce. Therefore, retrospective study was conducted to determine the proportion of IRD, changes in CD4+ T-cell count and possible risk factors of IRD during HAART.

## Methods

All HIV- infected subjects (≥18 years) who were seen at Zewditu memorial hospital HIV clinic, Addis Ababa, Ethiopia between September 1, 2005 and August 31, 2006 and who were naive to antiretroviral-treatment at the time they started HAART were retrospectively recruited. Patients who did not have adherence to HAART, who had previous antiretroviral exposure and subjects with incomplete clinical and laboratory data were excluded from the study. The hospital ethical review board and national ethical committee approved the protocol. Treatment initiation was in compliance with Ethiopian National Antiretroviral Treatment Guidelines [13]. The HAART was a combination of triple regimen with 2 nucleoside reverse-transcriptase inhibitors and a non- nucleoside reverse-transcriptase inhibitor.

After initiation of HAART, the study subjects were followed every 0.5, 1, 2, 3, 6, 9 and 12 months for any clinical complaints during the study period. Socio-demographic characteristics, previous clinical data, HAART, CD4+ T-cell count, white blood cell (WBC) count, hemoglobin (Hgb) level, alanine aminotransferase (ALT), aspartate aminotransferase (AST), and alkaline phosphatase (ALP) values were collected from computerized clinic records and patient charts at the initiation and 6 months of HAART time. Moreover, two senior physicians reviewed the patients' chart records to identify any clinical events (IRD) after commencing HAART (including date of onset, diagnostic methods, clinical history, etc).

With freshly collected blood samples, CD4+ T-cell count (cells/μl) was determined using FACSCount apparatus (Becton Dickinson, Sparks, MD., USA) following the manufacturer's protocol. WBC count (cells/μl), Hgb level (gm/dl), and ALT, AST and ALP (IU/L) values were also determined following the standard procedures [14].

Sputum or aspirates were collected from patients with clinical features suggestive of tuberculosis (TB).TB was diagnosed by smear microscopy to detect acid-fast bacilli (AFB), chest X-ray and/or Ultrasonic and clinical methods.

Diagnosis of cryptococcosis was based on laboratory and clinical features of the organism. Cerebrospinal fluid was examined microscopically for the detection of cryptococcal capsule using Indian ink following the standard procedure [14]. Toxoplasmosis was diagnosed by detecting immunoglobulin G using Enzyme-linked immunosorbent assay in addition to its clinical features and *Pneumocystis jirovecii *pneumonia (PCP) was identified using clinical and chest X-ray assessments and herpes zoster rash was diagnosed by clinical examination.

Diagnosis of IRD was based on previously published definitions [15-18]. In brief, subjects with HIV infection, low CD4+ T-cell count at baseline (most of the patients had < 90 CD4+ T-cell count/μl), and clinical symptoms consistent with inflammatory process after starting HAART considered to have developed IRD. Since viral load determination was not available in the country, it was not used as criterion to diagnose IRD. Patients who developed IRD were treated and managed as per routine clinical practice of the HIV clinic.

All data were entered and analyzed using SPSS version 15 packages (SPSS, Chicago,II., USA). Student's t-test and chi-square tests were employed for analysis of continuous and categorical data, respectively. Risk factors related to the development of IRD following HAART initiation were identified using binary logistic regression analyses and a *p *value of less than 0.05 was considered statistically significant.

## Results

A total of 1166 HIV- infected patients with mean ± SD age of 36 ± 9.3 year were included for this retrospective study. Majority of the patients were females (55.3%) and married (47.7%). Most of the study subjects had history of previous OIs and the predominant OIs investigated were herpes zoster rash (43.2%) followed by TB (27.6%). At time of HAART initiation, the patients were also diagnosed for OIs and the majority of the patients had candidiasis (37%) followed by TB (22.1%) (Table 1).

**Table 1:** Pattern of past opportunistic infections and opportunistic infections at time of HAART initiation in HIV- infected patients, at Zewditu Memorial Hospital, Addis Ababa, Ethiopia

Types of previous OIs	Frequency (%)	Types of OIs at time of HAART initiation	Frequency (%)
Herpes zoster	504 (43.2)	Candidiasis	431 (37.0)
PTB	236 (20.2)	PTB	150 ( 12.9)
EPTB	83 (7.1)	EPTB	64 (5.5)
DTB	3 (0.26)	DTB	44 ( 3.8)
Candidiasis	189 (16.2)	Toxoplasmosis	30 (2.6)
PCP	36 (3.1)	Herpes zoster	20(1.7)
Toxoplasmosis	35 (3.0)	PCP	7 (0.6)
Cryptococcosis	30(2.6)	Herpes simplex	2 (0.17)
Herpes simplex	14 (1.2)	**Total**	748 ( 64.2)
**Total**	1130(96.9%)		

***Keys***: OI, opportunistic infection; HAART, highly active antiretroviral therapy; PTB, pulmonary tuberculosis; EPTB, extra pulmonary tuberculosis; DTB, disseminated tuberculosis; PCP, Pneumocystis jirovecii pneumonia.

At time of HAART initiation, the mean ± SD of CD4+ T-cell count, WBC count and Hgb value of the total 1166 subjects, respectively, were 113.6 ± 71, 47671 ± 1824, 12 ± 2.4 and 28%, 23.6%, and 22.9% of the patients had an elevated AST, ALT, and ALP, respectively. According to the WHO AIDS clinical staging criteria, 55.7%, 31.7%, 11.4% and 1.2% of the patients, respectively, were classified under stage III, stage IV, stage II and stage I and the predominant HAART regimen given was 1b (combination of lamivudine, stavudine and efavirenz) followed by 1a (combinations of lamivudine, stavudine and nevirapine), 1d (combination zidovudine, lamivudine and efavirenz) and 1c (combination of zidovudine, lamivudine and nevirapine) for 34%, 23.3%, 22% and 20.6% of the patients, respectively at time of HAART initiation.

**Figure 1:**
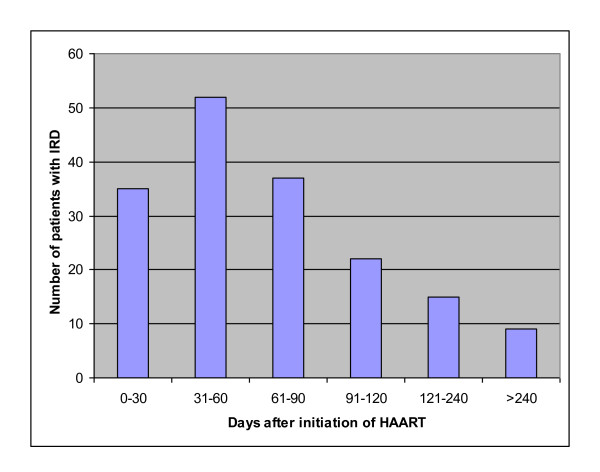
**Time (days) to diagnosis of IRD after initiation of HAART**.

One hundred seventy (14.6%) of the study subjects developed IRD. Table 2 shows the baseline characteristics of patients with and without IRD. The patients with IRD at HAART initiation were younger, had low CD4+ T-cell count, low WBC count and higher proportion of extra pulmonary tuberculosis(EPTB) (*P *< 0.05). However, there were no significantly differences in body weight, regimen, marital status, gender, pulmonary tuberculosis (PTB) and disseminated tuberculosis (DTB) between patients with and without IRD (*P *> 0.05). The interval between the start of HAART and the onset of IRD was variable and ranged from 11 to 329 days with a mean ± SD of 96 ± 89 days. Majority (72.9%) of the patients developed IRD within the first three months of HAART initiation (Figure 1).

**Table 2:** Baseline characteristics of study subjects at Zewditu Memorial Hospital, Addis Ababa, Ethiopia

Characteristic	Patients with IRD (n = 170)	Patients without IRD (n = 996)	P-value
Age(years),mean ± SD	33.9 ± 7.7	36.4 ± 9.5	0.001
Body weight (kg), mean ± SD	48.5 ± 7.3	50.7 ± 15.5	0.13
CD4+ (cells/μl), mean ± SD	84 ± 57.8	116 ± 69.4	<0.001
WBC(cells/μl),mean ± SD	4246 ± 1948	4814 ± 1729	<0.001
HAART regimens (%)			
Lamivudine/Stavudine/Efavirenz	31.7	34.4	0.50
Lamivudine/Stavudine/Nevirapine	27.1	22.7	0.21
Zidovudine/Lamivudine and Efavirenz	21.8	22.1	0.92
Zidovudine/Lamivudine/Nevirapine	19.4	20.8	0.68
Marital status (%)			
Single	28.8	31.9	0.42
Married	47.0	49.1	0.62
Divorced	11.8	9.2	0.30
Widowed	12.4	9.7	0.30
Gender (%)			
Male	49.4	43.9	0.18
Female	50.6	56.1	
Site of TB (%)			
PTB	29.4	33.7	0.27
EPTB	52.4	5.8	<0.001
DTB	4.7	3.9	0.63

Of the 170 IRD cases, 132 (77.6%) were new presentations (unmasking) and the 38 (22.4%) were due to worsening of a recognized infections (paradoxical). The most frequent OI associated with IRD in the study was TB (66.5%, 113/170) of which 47.8% (54/113), 46% (52/113) and 6.2% (7/113) were EPTB, PTB and DTB, respectively. Sixty nine point nine percent (79/113) of TB episodes were new presentations (PTB (57%, 45/79), EPTB (39.2%, 31/79) and DTB (3.8%, 3/79), and 30.1% (34/113) cases were due to worsening of a recognized infection (EPTB (67.6%, 23/34), PTB (20.6%, 7/34) and DTB (11.8%, 4/34)). Of the total TB/IRD patients 54% were positive for AFB and the source of specimens were from sputum (67%) and fine needle aspiration (33%).

IRDs other than TB/IRD were toxoplasmosis (12.9%, 22/170), herpes zoster rash (12.9%, 22/170), PCP (4.1%, 7/170), and cryptococcosis (3.5%, 6/170), and the unmasking infections involved were toxoplasmosis (22/170), herpes zoster rash (22/170), cryptococcosis (6/170) and PCP (3/170).

AIDS clinical stage shift was observed in 27.6% (47/170) of the IRD patients: 32 from clinical stage III to IV, 11 from clinical stage II to III, and 4 from clinical stage II to IV. Treatment shift was also observed in 21.2% (36/170) of the IRD patients, 7 from 1a to 1b, 6 from 1c to 1d, 5 from 1a to 1c, 5 from 1b to 1c, 4 from 1b to 1d, 3 from 1a to 1d, 3 from 1c to 1a, 2 from 1d to 1b, and 1 from 1b to 1a.

There was also a treatment shift in 6.6% (66/996) of the non IRD patients due to peripheral neuropathy (3.3% from 1b to 1d and 3.3% from 1a to 1c). Three point one percent (31/996) and 1.6% (16/996) of the non IRD patients had developed severe anemia (with a Hgb value of less than 6.9 gm/dl) and hepatotoxicity, respectively. Forty percent of the non-IRD patients had developed anemia with a Hgb value of less than or equal to 11 gm/dl.

**Figure 2:**
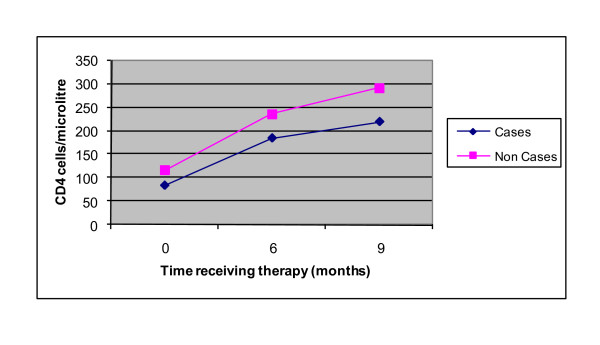
**Changes in CD4+ T-cell count for IRD (cases) and non IRD (non cases) patients versus number of months of treatment**.

For all study subjects, six months after initiation of HAART, the mean ± SD CD4+ T-cell count (230 ± 118), Hgb value (13.2 ± 3.8) and WBC count (6409 ± 1998), showed statistically significant elevation from the values at HAART initiation (*P *< 0.001). In addition, 34.5%, 31.4% and 26% of patients had significantly elevated values of AST, ALT and ALP respectively compared to the values at the initiation of HAART (*P *< 0.001). At nine months after initiation of HAART, both IRD (73%) and non IRD (27.4%) patients had a third CD4+ T-cell count with mean ± SD values of 220 ± 97.3 and 292 ± 145.6, respectively. The trend in CD4+ T-cell count changes versus number of months of treatment in patients with and without IRD is shown in Figure 2.

After commencement of HAART, laboratory values of  patients with and without IRD were compared and there were significant increases in CD4+ T-cell count, WBC count, ALT and AST in IRD and non IRD patients, and ALP and Hgb values  in non IRD patients (P <0.05) (Table 3).

**Table 3:** Laboratory values of patients with and without immune restoration disease before and after HAART commencement, at Zewditu Memorial Hospital, Addis Ababa, Ethiopia

Variables	Patients with IRD (n = 170)			Patients without IRD (n = 996)		
	**Mean (SD) values at baseline**	**Mean (SD) values after 6 months**	***P-value***	**Mean (SD) values at baseline**	**Mean (SD) values after 6 months**	***P-value***

CD4+ (cells/μl)	84 (57.8)*	185(94.8)	0.001	116 (69.4)	236(120)	0.001
WBC (cells/μl)	4246(1948)	5725 (3124)	0.001	4814 (1729)	6516(2072)	0.001
Hgb (gm/dl)	11.5(2.9)	12.1 (4.7)	0.400	12.2 (2.7)	13.9 (3.8)	0.001
AST (IU/L)	30.7(23.2)	37.8(28.8)	0.020	30(25.7)	43 (34.2)	0.001
ALT (IU/L)	25.3(20.2)	33.4(28.9)	0.003	25.1(23)	39 .2(34)	0.001
ALP (IU/L)	208(160)	213(167)	0.770	190 (149.9)	251 (212)	0.001

* Mean (SD); IRD, immune restoration disease; WBC, white blood cell; Hgb, hemoglobin; AST, aspartate aminotransferase; ALT, alanine aminotransferase; ALP, alkaline phosphatase.

Of the IRD patients 17.1% (29/170) needed to use systemic anti-inflammatory treatment to alleviate symptoms of IRD. There were eight deaths attributable to IRD and the causes of deaths were PTB, EPTB and DTB in 3, 3, and 2 of them, in that order. The mean ± SD baseline CD4+ T-cell count for these who died of IRD was 46 ± 17.6 and 19(11.2%) of IRD patients required hospitalization associated to their IRD occurrence.

Binary logistic regression was employed to assess if age, CD4+ T-cell count, WBC count, PTB, EPTB and DTB are possible risk factors for development of IRD. Low CD4+ T-cell counts (odds ratio [OR], 3.16, 95% confidence interval [CI], 2.19-4.58) and EPTB (OR, 7.7, 95% CI, 3.36-17.65) were found to be risk factors for development of IRD.

## Discussion

HAART improves immune function by suppressing HIV viral replication and increasing CD4+ T-cell counts [19]. Since its usage, IRD has been described in association with many concomitant infections such as mycobacterial, fungal and viral infections. In this retrospective study, from 1166 HIV/AIDS patients treated with HAART during the defined period of time, the proportion of IRD was 14.6% (170/1166). This finding is consistent with studies done elsewhere where the occurrence of IRD was between 10% - 25% [3,5,8,9,20,21]. In this study most of the IRD cases occurred within the first three months of HAART initiation, which is in agreement with prior reports [3,9,10].

Of the 170 IRD cases, 77.6% were new presentations, and 22.4% were due to paradoxical episodes. This report is in line with a previous study conducted elsewhere (8). Our finding of TB/IRD in majority of the IRD patients (9.7%) is in accordance with studies conducted in India, Thailand and Texas in which 7.6%, 12.6% and 14.4% IRD was caused by MTB [2,10,22]. However, our report is relatively low as compared with studies done in Thailand and Texas. Our low rate of MTB infection might be explained partly due to genetic polymorphism and racial differences of the study subjects [23]. And the nature of retrospective studies that may result differences in documenting and interpreting data in different settings also might play a role in variation of IRD reports.

In the study, 1.9% (22/1166) of the patients developed herpes zoster rash with mild and uncomplicated clinical manifestation. This finding is not consistent with a previous study in which a relatively high proportion of herpes zoster rash was indicated [8]. This variation may be due to the nature of our retrospective study. Soon after the initiation of HAART, it was observed that some patients presented with initial or recurrent episode of cryptococcal meningitis during the first weeks to months of therapy [24]. In the current study, cryptococcal meningitis was observed in 0.5% (6/1166) of the study subjects. This finding is in agreement with a previous study conducted somewhere else [8]. However, the report is low compared to a study conducted in France in which 8.3% cryptococcosis associated IRD was reported [25]. This discrepancy might be due to the difference in method employed for diagnosing of cryptococcosis.

In the study, 1.9% (22/1166) of the subjects developed toxoplasmosis and this figure is similar compared to the previous study [20]. In addition, 0.6% (7/1166) of the patients had developed PCP and this is comparable with a study conducted elsewhere [9].

In comparison with patients who did not develop IRD, the IRD patients had significantly low CD4+T- cell count and WBC count, and higher proportion of EPTB and younger age at baseline (*P *< 0.05). However, in binary logistic regression analyses low CD4+ T-cell count and EPTB were found to be risk factors for development of IRD. Previous studies also described that both low baseline CD4+ T-cell count and EPTB as the possible risk factors that were associated with the occurrence of IRD [22,26].

Thirty-one (3.1%, 31/996) patients had developed severe anemia with Hgb value below 6.9 gm/dl [27]. This might be due to the nature of some antiretroviral drugs which have myelosuppressive effect, especially with respect to the red blood cells which eventually lead to the development of anemia [28]. Sixteen (1.6%,16/996) of the study subjects also developed hepatotoxicity with three to five fold increments in serum levels of AST and ALT. This finding is in accordance with a study conducted by Becker [29]. This might be due to the direct effect of antiretroviral drugs, mainly nevirapine, that induce the development of hepatotoxicity [30]. Consistent with a previous report [8], in the present study we observed a 4.7% mortality rate after initiation of HAART among IRD patients.

## Conclusions

In this retrospective study, 14.6% of the patients had clinical deterioration (IRD) during immune recovery and eight deaths were attributable to IRD. Most IRDs were observed within the first three months of HAART initiation, primarily affecting patients with lower baseline CD4+ T-cell counts and the majority of IRD cases were TB/IRD. Low baseline CD4+ T-cell count and EPTB were associated with development of IRD. Therefore, strict following of patients during the first three months of HAART initiation and diagnosis of latent TB [31] would help to prevent complications related to TB/IRD.

## Competing interests

All authors declared that no competing interest. The content of this manuscript has not been published and/or submitted for consideration of publication elsewhere.

## Authors' contributions

KH: Study design, data collection, data analysis and write up; AK: Data analysis and write up; AM: Study design and write up; YW: write up. All authors read and approved the final manuscript.
